# Tucker3-PCovR: The Tucker3 principal covariates regression model

**DOI:** 10.3758/s13428-024-02379-3

**Published:** 2024-04-05

**Authors:** Elisa Frutos-Bernal, Laura Vicente-González, Jose Luis Vicente-Villardón

**Affiliations:** Department of Statistics, Universidad de Salamanca, Facultad de Medicina, Campus Miguel de Unamuno, Salamanca, 37007 Spain

**Keywords:** Multiway covariates regression, PCovR, Three-way, Tucker3 analysis, Biplot

## Abstract

In behavioral research, it is very common to have manage multiple datasets containing information about the same set of individuals, in such a way that one dataset attempts to explain the others. To address this need, in this paper the Tucker3-PCovR model is proposed. This model is a particular case of PCovR models which focuses on the analysis of a three-way data array and a two-way data matrix where the latter plays the explanatory role. The Tucker3-PCovR model reduces the predictors to a few components and predicts the criterion by using these components and, at the same time, the three-way data is fitted by the Tucker3 model. Both the reduction of the predictors and the prediction of the criterion are done simultaneously. An alternating least squares algorithm is proposed to estimate the Tucker3-PCovR model. A biplot representation is presented to facilitate the interpretation of the results. Some applications are made to empirical datasets from the field of psychology.

## Introduction

In our information-driven society, researchers are often faced with an ever-increasing influx of data. For example, it is not uncommon to find datasets that contain different types of information about the same group of individuals. This is commonly referred to as coupled data, defined as a collection of N-way N-mode data blocks, where each block shares at least one mode with another data block, as described in Wilderjans et al. (2009). To illustrate, consider the European Social Survey, where researchers can use different variables measured over time for a set of countries (such as media consumption, social trust, political engagement, etc.) together with additional non-temporal information about these countries. In this scenario, the coupled dataset consists of a three-way data array and a two-way data matrix that share one mode, namely the countries.

Multiblock data analysis presents two distinct categories of challenges. The first one is known as the multiblock component problem. Its primary objective is to identify sets of components that effectively capture the information present in all matrices simultaneously, essentially trying to uncover common information across multiple matrices. The second is referred to as the multiblock regression problem, which involves the use of multiple matrices either for prediction or as predictors. In this scenario, the matrices are used to predict each other or to predict a common outcome.

In the context of a multiblock component problem, the goal is to merge all available information to uncover the underlying structure within the interconnected data blocks. This is done by employing a global model that simultaneously analyzes the different data blocks, where each data block is summarized using an N-way N-mode decomposition model (such as PCA, PARAFAC, Tucker3 or Tucker2). In this N-way N-mode decomposition model, the data is decomposed into N component matrices, one for each of the N modes of the data, and potentially a linking array that captures the relationships among the components across the N modes, as described by Van Mechelen & Schepers (2007). In addition, constraints are introduced on the component matrices associated with the shared modes to account for the interconnections between data blocks. Several constraints can be considered, with the simplest being the identity constraint, where the component matrices are constrained to be identical for all data blocks sharing the same mode, as explained in Van Mechelen & Smilde (2010).

Some models have been proposed such as the PARAFAC-PCA (LMPCA) (Wilderjans et al., 2009). This model uses a simultaneous strategy to analyze a coupled dataset consisting of a real-valued three-way data array sharing a single mode with a real-valued two-way data matrix. In the PARAFAC-PCA model, a PARAFAC model (Hitchcock, 1927; Harshman, 1970; Carroll & Chang, 1970) is fitted to the three-way data and a PCA model (Pearson, 1901; Hotelling, 1933; Jolliffe, 2002) is fitted to the two-way data, so that the component matrix for the common mode is the same in both models. In this case, the two blocks of information play the same role.

In the domain of multiblock regression problems, the goal is to reduce the predictor variables to a limited number of components capable of predicting the dependent variables. Multiway multiblock covariates regression models (PCovR), as detailed in Smilde et al. (2000), represent a specific subset of multiblock regression models that simultaneously handle component reduction and prediction. In essence, these components are extracted in a way that simultaneously summarizes the predictor variables and optimally predicts the criterion scores. To elaborate, the predictor variables are transformed into a set of components that are linear combinations of the original variables, and then the criterion variables are regressed against these components, as explained in Gavaladze et al. (2021). This process involves minimizing a global criterion that depends on a weight parameter. This weight serves to signify the relative importance of reduction and prediction within the analysis, and ranges between 0 and 1. When the weight is set to 0, PCovR aligns with reduced rank regression (RRR, as discussed in Izenman (1975)), while a weight of 1 transforms it into principal component regression (PCR, as introduced in Jolliffe (1982)). Notably, in the latter case, the component matrix containing observation scores on the components remains identical in both decomposition methods.

In this paper, we will consider a specific scenario involving a coupled dataset. This dataset consists of a two-way data matrix that shares a common mode with a three-way data array, where the two-way block serves as an explanatory component for the three-way block. We present a multiway multiblock covariates regression model in which we decompose the three-way array based on the Tucker3 model. Additionally, we provide a comprehensive explanation of the algorithm used to conduct this analysis. Given the inherent complexity in interpreting the results generated by the Tucker3 model, we suggest the use of biplot representations to facilitate the understanding of the results.

The following sections of this paper are structured as follows. In Section 2, we first discuss the data structure and outline the preprocessing steps. Within this section, we introduce the Tucker3-PCovR model and explore its relationships with other models. In Section 3, we discuss the ALS algorithm used to estimate the model parameters. We also discuss various rank selection heuristics and address post-processing issues. Section 4 is dedicated to a comprehensive review of biplot representations tailored for three-way data, and also suggests the use of a useful representation called triplot to assess the relationship between the two matrices and make predictions. In Section 5, we put the Tucker3-PCovR model to practical use by applying it to empirical data sourced from the field of personality psychology. In this context, we also present a biplot representation of the results. Finally, Section 6 concludes the paper with some final remarks.

## Model

### Data and pre-processing

The Tucker3-PCovR analysis requires a $I \times L$ block of predictors $\textbf{X}$ (object by covariate) and a $I \times J \times K$ block of criteria $\mathbf {\underline{Y}}$ (object by attribute by source), both measured for the same *I* objects. In psychology, for example, the data array $\mathbf {\underline{Y}}$ can contain the reactions of a group of individuals to certain situations, while $\textbf{X}$ may contain the scores of the same group of individuals on a list of dispositions.

Before we can analyze our data, we need to prepare it so that it is in a format that is suitable for the analysis method we are using. This process is called data pre-processing. For two-way data matrices, we typically center and standardize the data. This means that we subtract the mean from each value and then divide by the standard deviation. This helps to make the data more comparable and easier to analyze. For three-way data arrays, we typically set the mean across the objects to 0 and the variance of each attribute to 1. This is similar to centering and standardizing two-way data matrices, but it takes into account the additional dimension of the data.

Data pre-processing is an important step in any data analysis project. By properly pre-processing our data, we can improve the quality of our results and make it easier to interpret our findings.

### Formulation of the model

The Tucker3-PCovR model decomposes the predictor matrix $\textbf{X}$ as follows:

1 $$\begin{aligned} \textbf{X} = \textbf{X}\textbf{W}_{X}\textbf{P}_{X}^{T}+\textbf{E}_{X} = \textbf{A}\textbf{P}_{X}^{T} + \textbf{E}_{X} \end{aligned}$$

where

2 $$\begin{aligned} \textbf{A} = \textbf{X}\textbf{W}_{X} \end{aligned}$$

is the $\left( I \times R_1 \right)$ component score matrix, containing the scores of the *I* observations on the $R_1$ components and $\textbf{P}_{X}$ is the $\left( L \times R_1 \right)$ loading matrix, which contains the loadings of the predictor variables on the components. Obviously, $R_1$ is (much) smaller than *L*. $\textbf{E}_{X}$ is a residuals matrix for $\textbf{X}$ and $\textbf{W}$ is the $\left( L \times R_1 \right)$ matrix of weights. Regarding the data array $\mathbf {\underline{Y}}$, its matricization $\textbf{Y}_{A}$ $\left( I \times JK\right)$ (Kiers, 2000) is regressed on the $R_1$ components:

3 $$\begin{aligned} \textbf{Y}_{A} = \textbf{A}\textbf{P}_{Y}^{T} + \textbf{E}_{A} \end{aligned}$$

where $\textbf{P}_{Y}$ $\left( JK \times R_1 \right)$ is the matrix containing the regression weights and $\textbf{E}_{A}$ is a residuals matrix for $\textbf{Y}_A$. Furthermore, $\textbf{P}_{Y}^{T}$ is decomposed using Tucker3 decomposition (Tucker, 1966):

4 $$\begin{aligned} \textbf{P}_Y^{T} = \textbf{G}_A \left( \textbf{C} \otimes \textbf{B}\right) ^{T} \end{aligned}$$

where $\otimes$ denotes the Kronecker product, $\textbf{G}_A$ is the $\left( R_1 \times R_2\right.$ $\left. R_3\right)$ matricization of the core array $\mathbf {\underline{G}}$, $\textbf{B}$ is the $\left( J \times R_2\right)$ component score matrix for the second mode and $\textbf{C}$ is the $\left( K \times R_3\right)$ component score matrix for the third mode. Note that $\left( R_1, R_2, R_3 \right)$ is the complexity of the Tucker3 model.

So, $\textbf{Y}_A$ could be written as:

5 $$\begin{aligned} \textbf{Y}_A = \textbf{A}\textbf{G}_A \left( \textbf{C} \otimes \textbf{B}\right) ^{T} + \textbf{E}_A \end{aligned}$$

Since both the PCovR solution and Tucker3 decomposition have rotational freedom, also the Tucker3-PCovR solution is not unique.

### Relation to other models

The Tucker3-PCovR model is related to other multiway multiblock models, both within and outside the family of multiway multiblock regression models (Smilde & Kiers, 1999). Within the family of multiway multiblock regression models, this model is a special case where the three-way data block is decomposed according to a Tucker3 decomposition. In turn, multiway multiblock regression models are a generalization of the PCovR model (de Jong & Kiers, 1992).

Furthermore, multiway multiblock regression models are closely related to the family of multiway multiblock component models (Smilde et al., 2000). The main difference lies in the role that data blocks play in the analysis. In a multiway multiblock component model, all data blocks play the same role. In a multiway covariates regression model, some data blocks play the role of predictor blocks, while the other block(s) play the role of criterion block(s). An example of a multiway multiblock component model related to the Tucker3-PCovR model is the LMPCA (Wilderjans et al., 2009).

## Data analysis

### Aim

In PCovR analysis, the reduction of the predictor variables to components and the prediction of the criterion variables by these components is performed simultaneously by minimizing the following loss function:

6 $$\begin{aligned} L\left( {\textbf {A}}, {\textbf {B}}, {\textbf {C}}, {\textbf {G}}_A | \alpha , {\textbf {X}}, {\textbf {Y}}_A \right) = \alpha \frac{\Vert {{\textbf {X}} - {\textbf {A}}{} {\textbf {P}}_{X}^{T}}\Vert ^{2}}{\Vert {{\textbf {X}}}\Vert ^2} + \left( 1- \alpha \right) \frac{\Vert {{\textbf {Y}}_A - {\textbf {A}}{} {\textbf {P}}_{Y}^{T}}\Vert ^{2}}{\Vert {{\textbf {Y}}_A}\Vert ^2}= & {} \nonumber \\ \alpha \frac{\Vert {{\textbf {X}} - {\textbf {A}}{} {\textbf {P}}_{X}^{T}}\Vert ^{2}}{\Vert {{\textbf {X}}}\Vert ^2} + \left( 1- \alpha \right) \frac{\Vert {{\textbf {Y}}_A - {\textbf {A}}{} {\textbf {G}}_A \left( {\textbf {C}} \otimes {\textbf {B}}\right) ^{T}}\Vert ^{2}}{\Vert {{\textbf {Y}}_A}\Vert ^2} \end{aligned}$$

where $\alpha$ is a weighting parameter which specifies to what degree reduction and prediction are emphasized, $0 \le \alpha \le 1$. Matrices $\textbf{A}$, $\textbf{B}$, $\textbf{C}$ are the component matrices for the first, second, and third modes, respectively, and $\mathbf {G_A}$ is the matricization of the core array $\mathbf {\underline{G}}$. When $\alpha =0$, all emphasis is on predicting $\mathbf {\underline{Y}}$ and PCovR analysis becomes equivalent to reduced rank regression (RRR) (Aldrin, 2006) and when $\alpha =1$, all emphasis is on explaining $\textbf{X}$ and PCovR analysis becomes equivalent to principal component regression (PCR) (Jolliffe, 1982). When $\alpha$ takes high values, the focus is on strong components (i.e., components which explain a lot of variance in $\textbf{X}$) while low $\alpha$ values focus on relevant components (i.e., components which explain a lot of variance in $\mathbf {\underline{Y}}$) (Gavaladze et al., 2021).

Note that the loss function above can be rewritten in the following way (Smilde & Kiers, 1999):

7 $$\begin{aligned} L = \frac{\alpha }{{\Vert {{\textbf {X}}}\Vert ^2}}{\Vert {{\textbf {X}} - {\textbf {A}}{} {\textbf {P}}_{X}^{T}}\Vert ^{2}} + \frac{\left( 1- \alpha \right) }{{\Vert {{\textbf {Y}}_A}\Vert ^2}} {\Vert {{\textbf {Y}}_A - {\textbf {A}}{} {\textbf {P}}_{Y}^{T}}\Vert ^{2}}= & {} \nonumber \\ \beta {\Vert {{\textbf {X}} - {\textbf {A}}{} {\textbf {P}}_{X}^{T}}\Vert ^{2}} + \left( 1-\beta \right) {\Vert {{\textbf {Y}}_A - {\textbf {A}}{} {\textbf {P}}_{Y}^{T}}\Vert ^{2}}= & {} \nonumber \\ \Vert {{\textbf {Q}} - {\textbf {A}}{} {\textbf {S}}\Vert ^T}^2 \end{aligned}$$

where

8 $$\begin{aligned} {\textbf {Q}} = \sqrt{\beta }{} {\textbf {X}} \mid \sqrt{1-\beta }{} {\textbf {Y}}_A \end{aligned}$$

9 $$\begin{aligned} {\textbf {S}}^T\!=\!\sqrt{\beta }{} {\textbf {P}}_X^T \mid \sqrt{1-\beta }{} {\textbf {P}}_Y^T \!=\! \sqrt{\beta }{} {\textbf {P}}_X^T \mid \sqrt{1-\beta }{} {\textbf {G}}_A \left( {\textbf {C}} \otimes {\textbf {B}}\right) ^{T} \end{aligned}$$

and

10 $$\begin{aligned} \beta = \frac{\alpha {\Vert {{\textbf {Y}}_A}\Vert ^2}}{\alpha {\Vert {{\textbf {Y}}_A}\Vert ^2+\left( 1- \alpha \right) \Vert {{\textbf {X}}}\Vert ^2}} \end{aligned}$$

### Algorithm

The parameters of the Tucker3-PCovR model are estimated using an alternating least squares (ALS) algorithm (Kroonenberg & De Leeuw, 1980). In this algorithm, starting values for all model parameters are obtained, and then the component matrices $\textbf{A}$, $\textbf{B}$, $\textbf{C}$, and $\textbf{P}_X^T$ and the core array $\underline{\textbf{G}}$ are alternately re-estimated conditionally upon the other parameters until a convergence criterion is satisfied (ten Berge, 1993).

There are two main options for initializing the component matrices in the Tucker3-PCovR model: random initialization and rational initialization. For random initialization, each entry of the component matrices is sampled from a standard normal distribution. For rational initialization, the component matrices are initialized as follows. Suppose that $\left( R_1, R_2, R_3\right)$ is the complexity of the Tucker3 model and $\textbf{Y}_A$, $\textbf{Y}_B$, $\textbf{Y}_C$ are the $\left( I \times JK \right)$, $\left( J \times IK \right)$, $\left( K \times IJ \right)$ matricized versions of $\mathbf {\underline{Y}}$ where $\mid$ denotes matrix concatenation. Rational initial estimates of $\textbf{B}$ and $\textbf{C}$ are obtained by taking the eigenvectors associated with the $R_2$, $R_3$ largest eigenvalues of $\textbf{Y}_B \textbf{Y}_B^T$ and $\textbf{Y}_C \textbf{Y}_C^T$, respectively. Note that this procedure ensures that the initial $\textbf{B}$ and $\textbf{C}$ are orthonormal. $\textbf{A}$ is obtained by taking the eigenvectors associated with the $R_1$ largest eigenvalues of $\left( \textbf{X} \mid \textbf{Y}_A\right) \left( \textbf{X} \mid \textbf{Y}_A\right) ^T$. Once the initial estimates of $\textbf{A}$, $\textbf{B}$ and $\textbf{C}$ are obtained, the initial estimates of $\mathbf {\underline{G}}$ and $\textbf{P}^T_X$ are obtained by regression.

Once the complete set of initial estimates of the model parameters has been obtained, the Tucker3-PCovR algorithm performs an iterative process until a pre-specified stopping criterion is reached. In each iteration, the component matrices $\textbf{A}$, $\textbf{B}$, and $\textbf{C}$, and the core matrix $\underline{\textbf{G}}$, are recalculated conditionally upon the other parameters. Specifically, the following steps are performed in each iteration:

- Fixing $\textbf{B}$, $\textbf{C}$, $\underline{\textbf{G}}$, and $\textbf{P}_X^T$, the matrix $\textbf{A}$ is re-estimated.
- Fixing $\textbf{A}$, $\textbf{C}$, and $\underline{\textbf{G}}$, the matrix $\textbf{B}$ is re-estimated.
- Fixing $\textbf{A}$, $\textbf{B}$, and $\underline{\textbf{G}}$, the matrix $\textbf{C}$ is re-estimated.
- Fixing $\textbf{A}$, $\textbf{B}$, and $\textbf{C}$, the core matrix $\underline{\textbf{G}}$ and the projection matrix $\textbf{P}_X^T$ are updated by solving a multivariate linear regression problem (Kroonenberg & De Leeuw, 1980; Smilde et al., 2004; Andersson & Bro, 1998; Bro & Andersson, 1998).

After each iteration, the loss function is recalculated from the new values of the model parameters. When there is a substantial decrease in the loss function, a new iteration is performed. Otherwise, the algorithm stops and the values of the last iteration are taken as the final values of the model parameters.

This iterative process is repeated until a convergence criterion is satisfied, such as when the change in the loss function is below a certain threshold or when a maximum number of iterations is reached. This process works by gradually improving the estimates of the model parameters until a convergence criterion is satisfied. In each iteration, one of the model parameters is re-estimated while keeping the other parameters fixed. This allows the algorithm to gradually adjust the model parameters to minimize the loss function.

Although the convergence of the Tucker3-PCovR algorithm is guaranteed, this does not mean that it cannot end up in a local rather than the global optimum. To try to avoid this, it is recommended to use a multi-start procedure, in which the algorithm is run multiple times, each time with different random or pseudo-random initialized parameter values. Pseudo-random initial values can be obtained by slightly perturbing a rationally initialized solution (Ceulemans et al., 2007). The solution with the lower value of the loss function is kept. A more detailed description of the algorithm can be found in Appendix A.

### Model selection

To select a Tucker3-PCovR solution, it is necessary to determine the $\alpha$ value and the complexity of the Tucker3 model $\left( R_1, R_2, R_3\right)$.

Given $\alpha$ the complexity of the model can be determined by means of a scree test (Ceulemans & Kiers, 2009) or by cross-validation (Vervloet et al., 2016; Hastie et al., 2009). When performing a scree test different analyzes with different complexity are performed, taking into account the rank of the model values between $\left( 1, 1, 1 \right)$ and $\left( R_1^{Max},R_2^{Max}, R_3^{Max}\right)$. The model that has a good balance between model fit ($f_i$) and model complexity ($c_i$) is selected. Model fit can be quantified by the loss function value. To measure model complexity, the total number of components (i.e., $c_i = R_1 + R_2 + R_3$) is used. Next, the CHULL procedure (Ceulemans & Kiers, 2006, 2009; Wilderjans et al., 2013) can be used to determine the optimal rank. The CHULL method consists of the following steps: (1) selecting those models which are located on the boundary of the convex hull from the plot obtained by plotting the complexity versus fit of all the valid models and (2) identifying for which model there is a good balance between its complexity and its fit. This is done by calculating the *st*-ratio for each model:

11 $$\begin{aligned} st_i = \frac{\left( f_{i-1}-f_i\right) /\left( c_i-c_{i-1}\right) }{\left( f_{i}-f_{i+1}\right) /\left( {c_{i+1}-c_i}\right) } \end{aligned}$$

and the model with the highest *st*-value is selected. Some guidelines on how to choose the alpha value can be found in Vervloet et al. (2013).

### Rotational freedom

Since PCovR and Tucker3 decomposition have rotational freedom, the Tucker3-PCovR decomposition is not unique either. For example, the component matrices **A**, **B**, and **C** obtained by the Tucker3 decomposition can be rotated as long as the core array $\underline{{\textbf {G}}}$ (and in the case of **A** also ${\textbf {P}}_X$) is counterrotated. In addition, the matrices **A** and ${\textbf {P}}_X$ can be rotated as long as the core array and the other non-rotated matrix (${\textbf {P}}_X$ and **A** respectively) are compensated. Researchers can take advantage of this rotational freedom to improve the interpretability of the solution obtained. There are several strategies to do this. The first option is to rotate the component matrices, for example using a varimax rotation (Kaiser, 1958), and then counterrotate the core array. In this case, more easily interpretable component matrices are obtained at the expense of having a core array with many large numbers (i.e., complex interactions between the components). The second option is to rotate the core array and then counterrotate the component matrices (Kiers, 1998a, b, 1992). This ensures that the core array has only a few large values, indicating the most important interactions between the components, but the components may be more difficult to interpret. Finally, it is possible to simultaneously rotate the core array and the component matrices in such a way that a criterion is optimized that balances the complexity of the component matrices and the core array (Kiers, 1997). It is important to note that the Tucker3-PCovR model is still identified after permutation and/or reflection of the components, even after the freedom of rotation is fixed.

Rotation of component matrices in a Tucker3 PCovR model may be advisable in certain scenarios to facilitate a more interpretable and meaningful representation of the underlying relationships. Matrix rotation is particularly useful when the original factor loadings are complex or difficult to interpret. The application of rotation aims to simplify the structure of the components, making it easier to identify and understand the relationships between the variables. This is particularly relevant when dealing with large datasets or models with many components, where rotation helps to achieve a more concise and insightful representation of the underlying patterns.

## Biplot representation

The interpretability of the results of a Tucker3 analysis can often be challenging due to the complexity of interpreting the connections between the modes contained within the core array. One potential solution to address this challenge is the use of a biplot representation, as suggested by Gabriel (1971). Biplots serve the purpose of creating a low-dimensional visual representation of a data matrix. This representation allows us to visualize the relationships between individuals and variables, as well as the relationships between both groups.

### A general definition of a biplot

For a data matrix $\textbf{X}$ of size $I \times L$, the biplot graphical representation involves the placement of markers (vectors) $\textbf{a}_1, \textbf{a}_2, \ldots , \textbf{a}_I$ for the rows and $\textbf{b}_1, \textbf{b}_2, \ldots , \textbf{b}_L$ for the columns of $\textbf{X}$ in such a way that their inner product:

12 $$\begin{aligned} \hat{x}_{ij} = \textbf{a}_i^T \textbf{b}_j \end{aligned}$$

closely approximates the element $x_{ij}$ of $\textbf{X}$. Vectors are usually two- or three-dimensional, which means that we can easily draw them on a piece of paper or computer screen.

The most classical biplot representation is derived from approximating the data matrix $\textbf{X}$ of rank *r* with a lower-rank matrix $\textbf{X}$ through singular value decomposition (SVD), $\textbf{X} = \textbf{U}\varvec{\Delta }\textbf{V}^T$, where $\textbf{U}$ and $\textbf{V}$ are matrices of orthonormal singular vectors satisfying $\textbf{U}^{T} \textbf{U} = \textbf{I}$ and $\textbf{V}^{T} \textbf{V} = \textbf{I}$, and $\varvec{\Delta }$ is a diagonal matrix containing the singular values.

For the biplot visualization, the previous factorization is used, allowing us to express $\textbf{X}$ as $\textbf{X} = \textbf{A}^*\textbf{B}^{*T}$, where $\textbf{A}^* = \textbf{U}\varvec{\Delta }^s$ and $\textbf{B}^* = \textbf{V}\varvec{\Delta }^{1-s}$. The scalar *s*, typically ranging between 0 and 1, plays a crucial role in determining the type of biplot: when $s = 0$, it is referred to as a GH-biplot, and when $s = 1$, it is known as a JK-biplot. These biplots are closely related to principal component analysis or factor analysis, two of the most popular techniques for data analysis.

From a broader perspective, any matrix’s decomposition into the product of two other matrices with lower ranks can be employed as a basis for creating a biplot. Such decompositions appear in many other multivariate techniques such as canonical analysis, correspondence analysis and many others (Gower et al., 2011) and not always as the result of an SVD.

**Fig. 1:**
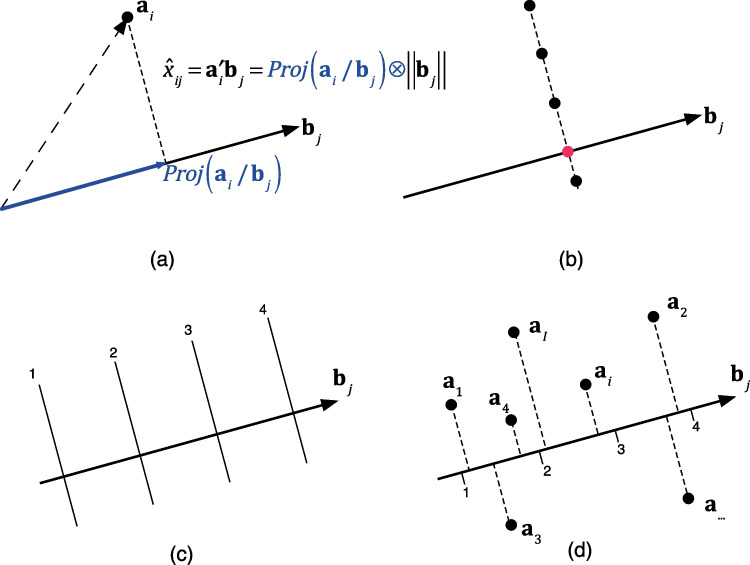
Biplot approximation: **a** Inner product of the row and column markers approximates (predicts) the element of the matrix. **b** The set of points predicting the same value are all on a straight line perpendicular to the direction defined by the column marker ${{\mathbf{{b}}_j}}$. **c** Points predicting different values are on parallel lines. **d** The variable direction can be supplemented with scales to visually obtain the prediction

The geometric representation of a biplot is shown in Fig. 1, and it remains independent of the specific decomposition method used. Derived from the inner product described in Eq. 1, points that predict the same value lie on a straight line perpendicular to the biplot marker ${\mathbf{{b}}_j}$ (as shown in Fig. 1, (a, b). Different predicted values are situated on parallel lines (Fig. 1, (c–b). To improve the usefulness of the biplot, graded scales can be incorporated, allowing for the estimation of approximate values for the matrix entries by projecting the row markers onto the variable directions with these graded scales (Fig. 1, (d)). The calculations required to obtain the scale markers are straightforward.

We assume that the columns are centered to have zero mean and then no constant needs to be fitted. Note that the point that predicts 0 for all the variables is the origin, i.e., it predicts the mean of each variable. It is convenient to label the graded scaled with the initial rather than the transformed values. For example, if the variable is centered, we could use $\mu + {{\bar{x}}_i}$ to the label of the point $\mu$.

To interpret a general biplot, the following factors should be considered:

- Proximity of points: The proximity of points to each other indicates their degree of similarity or dissimilarity. Points closer together are more similar, while points further apart are more dissimilar.
- Angle between vectors: The angle between two vectors indicates the relationship or correlation between the corresponding variables. A small angle suggests a strong positive correlation, while a large angle implies a weaker or negative correlation.
- Vector lengths approximate the variability of the variables they represent.
- Inner products approximate the elements of the matrix.

### Biplots for three-way data

When discussing three-way data, two types of biplots can assist in the interpretation of analysis results: the joint biplot and the interactive biplot (Carlier & Kroonenberg, 1996).

#### Joint biplots

Joint biplots are constructed using the following decomposition:

13 $$\begin{aligned} y_{ijk} = \sum _{r=1}^{R_3} c_{kr} \left[ \sum _{p=1}^{R_1}\sum _{q=1}^{R_2} a_{ip}b_{jq}g_{pqr} \right] = \sum _{r=1}^{R_3} c_{kr} d_{\left( ij \right) r} \end{aligned}$$

where $d_{\left( ij \right) r}$ corresponds with the term in square brackets.

To construct a joint biplot, it is essential to work with the matrix $\textbf{D}_r = \textbf{A}\textbf{G}_r\textbf{B}^T = \textbf{A}^*_r\textbf{B}^{*T}_r$. This involves performing a singular value decomposition (SVD) for each value of *r* on the core slice $\textbf{G}_{r} \left( R_1 \times R_2\right)$: $\textbf{G}_{r} = \textbf{U}_{r}\varvec{\Delta }_{r}\textbf{V}_{r}^T$ with $\textbf{U}_{r}^{T} \textbf{U}_r= \textbf{I}$ and $\textbf{V}_{r}^{T} \textbf{V}_r= \textbf{I}$. Consequently, $\textbf{D}_r = \textbf{A}\textbf{U}_r\varvec{\Delta }_r\textbf{V}_r^T\textbf{B}^T$.

Next, the orthonormal left singular vectors $\textbf{U}_r$ and the orthonormal right singular vectors $\textbf{V}_r$ are combined with $\textbf{A}$ and $\textbf{B}$, respectively. The diagonal matrix $\varvec{\Delta }_{r}$, which contains the singular values, is divided between them as follows: $\textbf{A}^{*}_{r} = \left( \frac{I}{J}\right) ^{1/4}\textbf{A}\textbf{U}_{r}\varvec{\Delta }_{r}^{1/2}$ and $\textbf{B}^{*}_{r} = \left( \frac{J}{I}\right) ^{1/4}\textbf{B}\textbf{V}_{r}\varvec{\Delta }_{r}^{1/2}$ (the fourth-root fractions are introduced to account for the different number of levels in the two-component matrices). Consequently, the columns of the fitted component matrices, $\textbf{A}^{*}_{r}$ and $\textbf{B}^{*}_{r}$, represent the axes of the joint biplot.

Thus, when dealing with joint biplots, a crucial decision must be made: the selection of both the display mode and the reference mode.

A notable drawback of a joint biplot is that its interpretation requires an examination of the reference mode to determine the sign of the interaction. This makes interpretation difficult. For our particular problem, joint biplots are probably less useful.

#### Interactive biplots

The second category of biplot is called an interactive biplot and is also based on the Eq. 13. In this biplot, a single marker is used to represent each possible combination of variables originating from two of the modes. For example, if the second and third modes are combined, there will be $J \times K$ column markers. In this context, the row and column markers are $\textbf{A}^* = \textbf{A}$ and $\textbf{B}^* = \textbf{G}_A \left( \textbf{C} \otimes \textbf{B}\right) ^{T}$. This is basically the same as the Eq. 3 with $\textbf{B}^* = \textbf{P}_{Y}^{T} = \textbf{G}_A \left( \textbf{C} \otimes \textbf{B}\right) ^{T}$. Then we have a set of markers $\textbf{a}^*_1, \textbf{a}^*_2, \ldots , \textbf{a}^*_I$ for the first mode and a set $\textbf{b}^*_{1(1)}, \ldots , \textbf{b}^*_{1(K)}, \ldots , \textbf{b}^*_{J(1)}, \ldots , \textbf{b}^*_{J(K)}$ for the second and third interactively coded modes.

The interpretation of interactive biplots is identical to that of two-dimensional biplots, as we also obtain a (close) approximation of the values in the data matrix.

A notable disadvantage of this type of biplot is the potentially large number of column markers that can occur when dealing with large arrays, which can make them difficult to interpret. This type of biplot is most useful when the product $J \times K$ remains manageable in size. On the other hand, its main advantage lies in its ability to simultaneously display all the information associated with the three-way data array.

One possible approach to simplify interpretation is to focus on representing a single attribute across all the sources or representing all the attributes for a single source.

### Interactive biplots for responses and predictors: Triplots

The biplots described above are good for understanding how the dependent variables relate to each other, but not so good for predicting the values of the dependent variables from the values of the independent variables, or the relationships between the two sets. For this last purpose, several other biplots can be plotted.

#### Simultaneous representation of predictors and responses

The first is a representation that combines the representations for predictors and responses as follows. Equation 1 defines a biplot for the predictors $\textbf{X}$, sharing the $\textbf{A}$ coordinates with the previous one. Consequently, the coordinates in $\textbf{P}_X$ can be incorporated into the existing biplot, allowing the simultaneous representation of individuals ($\textbf{A}$), predictors ($\textbf{P}_X$), and responses ($\textbf{P}_Y$). This combined graphical representation is called a “triplot”. It essentially combines two biplots, the first showing the structure of the predictors and the second showing the responses. Both biplots focus on illustrating the relationships between these two sets of variables as in Ter Braak (1990) for two way matrices. The angles between variables of different sets are also interpreted in terms of their correlation.

#### Biplot representation of the regression weights

If we combine the Eq. 3 with the Eq. 2 we get:

14 $$\begin{aligned} \textbf{Y}_{A} = \textbf{XW}_X \textbf{P}_{Y}^{T} + \textbf{E}_{A} \end{aligned}$$

thus

15 $$\begin{aligned} \textbf{Z} = \textbf{W}_X \textbf{P}_{Y}^{T} \end{aligned}$$

define a set of regression weights on the original variables that can be plotted on a biplot to help assess the importance of each predictor in explaining each response and the direction of its effect. The markers for the predictors are now represented by the rows of $\textbf{W}^* = \textbf{W}_X$, and the markers for the responses are the rows of $\textbf{B}^*$ as defined previously. The inner product $\hat{z}_{lj(k)} = \textbf{w}^{*T}_l \textbf{b}^*_{j(k)}$ represents the weight of the *l*-th variable in predicting the *j*-th attribute at the *k*-th source. For example, projecting all the markers $\textbf{w}^*_1, \textbf{w}^*_2, \ldots , \textbf{w}^*_L$ onto $\textbf{b}^*_{j(k)}$ can help determine the relative importance of each variable on the attributes and the direction of the effect. Positive and negative predictions correspond to positive and negative effects. This biplot is similar to the partial least squares (PLS) proposals in Oyedele & Lubbe (2015) or Vicente-Gonzalez & Vicente-Villardon (2022).

#### Interpolation and prediction biplots

All the described biplots are “prediction biplots” because the inner product of two vectors predict some value (the observed value of the data matrix or some regression coefficient). There is another kind of biplot that could be useful in this context, the interpolation biplot for the predictors, combined with the prediction biplot for the responses. That allows for the projection of new supplementary individuals on the representation, using a set of values for the predictors, and then predict the values of the responses from the biplot scores.

We have that $\textbf{W}=\textbf{W}_X$ contains the vectors to interpolate a new point on the representation. Suppose we have a new observation ${\textbf{x}}=(x_1, \dots , x_J)^T$, using Eq. 2 we can project the new observation onto the biplot with

16 $$\begin{aligned} {\textbf{a}}={\textbf{x}}^T{\textbf{W}}=\sum _{l=1}^L x_j {\textbf{w}}_l \end{aligned}$$

**Fig. 2:**
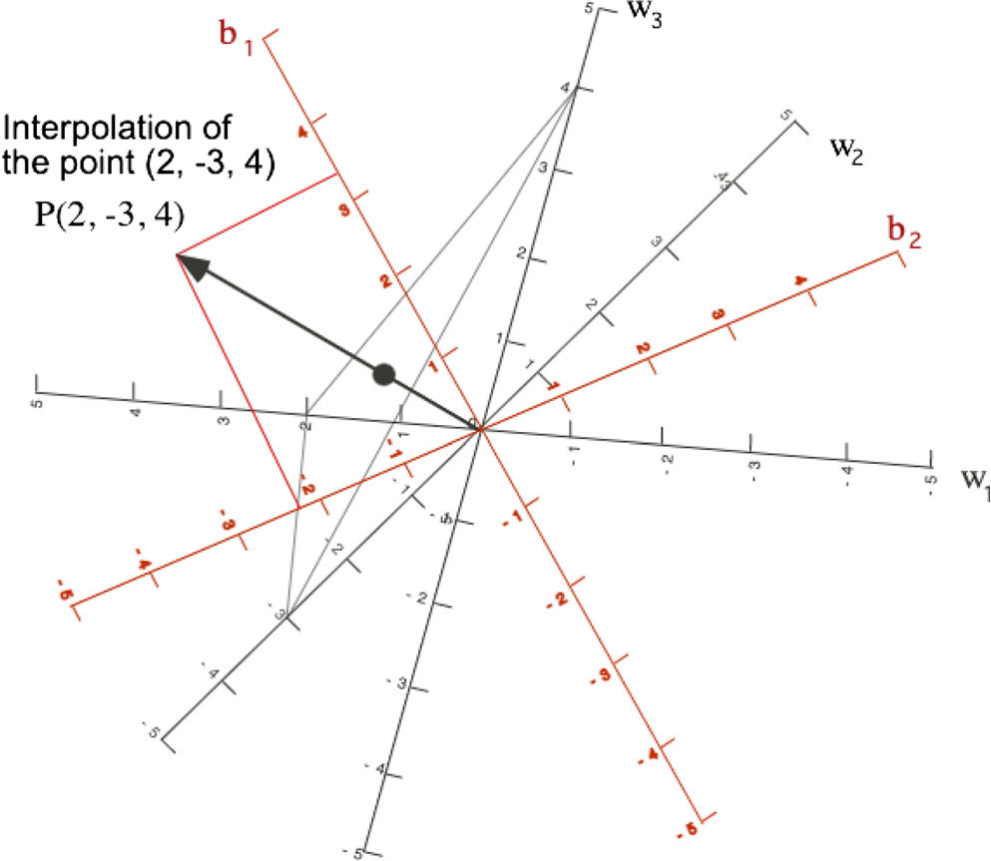
Interpolation biplot with three independent variables (*black*), and prediction biplot for the responses (*red*)

That is a weighted sum of the vectors ${\textbf{w}}_l$ using the observed values ${\textbf{x}}=(x_1, \dots , x_J)^T$ as weights. The graphical interpretation of the interpolation is shown in Fig. 2. The sum of the vectors is calculated here using the centroid multiplied by the number of points (the position of the new point is the end of the arrow). Once the point is interpolated we can obtain a prediction of the responses by projecting it on the directions for the dependent variables. See, for example, Gower (1996) or Gower et al. (2011).

The directions are given by the ${\textbf{w}}$’s and scales are easily placed. To find the marker for a fixed value $\mu$, on the direction of vector ${\textbf{w}}_l$ we look for the point (*x*, *y*)

17 $$\begin{aligned} x = {\mu \;{w_{l1}}};\quad \quad y = {\mu \;{w_{l2}}} \end{aligned}$$

The analytic interpolation is probably more useful than the geometric interpolation. We have added it here to illustrate the process.

## Illustrative application

An important goal in the field of psychology is to analyze the relationship between the way people react to a particular situation and the dispositions or traits they have (Van Coillie et al., 2006). It seems reasonable to think that personality traits can explain reactions to certain situations. To this end, we will perform a Tucker3-PCovR analysis on a pairwise dataset that contains, on the one hand, the reactions of a group of people to certain situations and, on the other hand, their personality traits.

### First example

The first example consists of a hypothetical 8 persons $\times$ 7 emotions $\times$ 6 situations data array and an 8 persons $\times$ 10 dispositions data matrix (Wilderjans et al., 2009).

The three-way data array was pre-processed in such way that the scores were mean-centered across persons and the scores for each response were normalized. The disposition data matrix was also pre-processed by standardizing.

**Fig. 3:**
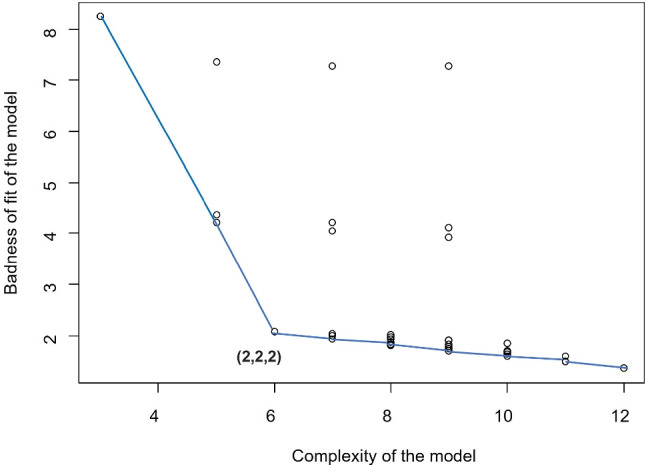
Chull plot of the complexity of the model versus the loss function value for all Tucker3-PCovR estimated models with $\alpha$=0.5

Tucker3-PCovR analysis was performed on the coupled dataset for all valid ranks between $\left( 1,1,1\right)$ and $\left( 4,4,4\right)$ and $\alpha = 0.50$ (equal importance for degree reduction and prediction). The Chull model selection procedure was used to select from the estimated models a solution that best balances model fit and model complexity. The Chull model was used with the value of the loss function as the (mis)fit value and the total number of components as the complexity value. Based on the results obtained (Fig. 3) the solution $\left( 2,2,2 \right)$ was chosen.

**Table 1:** Component values for the situations, resulting from the Tucker3 PCovR (values exceeding 0.4 in absolute value are in bold)

Situation	Label	Comp 1	Comp2
Quarrelling with someone	S1	$\mathbf {-0.56}$	0.16
Partner leaves you	S2	$\mathbf {-0.67}$	−0.04
Someone is telling lies about you	S3	$\mathbf {-0.48}$	−0.04
Giving a bad speech	S4	−0.02	**0.56**
Failing a test	S5	0.01	**0.63**
Writing a bad paper	S6	0.09	**0.51**

Looking at the scores of the situation component (Table 1), the first component can be interpreted as an interpersonal dimension because situations in which there is a confrontation with others load high on this component (quarrelling with someone, someone is telling lies about you, partner leaves you). In contrast, the second component can be interpreted as an intrapersonal dimension because situations such as giving a bad speech, failing a test, and writing a bad paper score high on this component.

Regarding the response component scores (Table 2), reactions such as other anger, shame, sorrow, and love score on the first component, the first three negatively while the last one positively. Secondly, guilt and self-anger (emotions that force a person to confront himself) score high on the second component.

When interpreting the person component scores (Table 3), it can be seen that all persons score positive on the first component, the first three persons score highly negative on the second component, while the last four persons score highly positive on the second component. Note that person4 scores close to 0 on the second component. There is a similarity between person1, person2, and person3 on the one hand and person5, person6, person7, and person8 on the other.

**Table 2:** Component values for the response scales, resulting from the Tucker3-PCovR (values exceeding 0.4 in absolute value are in bold)

Response	Label	Comp 1	Comp2
Other anger	RP1	$\mathbf {-0.47}$	−0.21
Shame	RP2	$\mathbf {-0.52}$	−0.22
Love	RP3	**0.44**	0.20
Sorrow	RP4	$\mathbf {-0.52}$	0.26
Fear	RP5	−0.03	−0.06
Guilt	RP6	−0.15	**0.61**
Self-anger	RP7	−0.11	**0.65**

**Table 3:** Component values for the person scales, resulting from the Tucker3-PCovR

Person	Comp 1	Comp2
Person 1	0.36	−0.38
Person 2	0.32	−0.45
Person 3	0.38	−0.33
Person 4	0.61	0.05
Person 5	0.20	0.34
Person 6	0.26	0.36
Person 7	0.29	0.42
Person 8	0.26	0.35

From the Table 4, it can be seen that dispositions which characterize people who are more other-oriented (fear to be refused, kindness, importance of others’ judgments, altruism) have positive scores on the second component, while those which characterize people who are more self-oriented (being strict to oneself, low self-esteem, conscientiousness, depression) have negative scores on the second component.

**Fig. 4:**
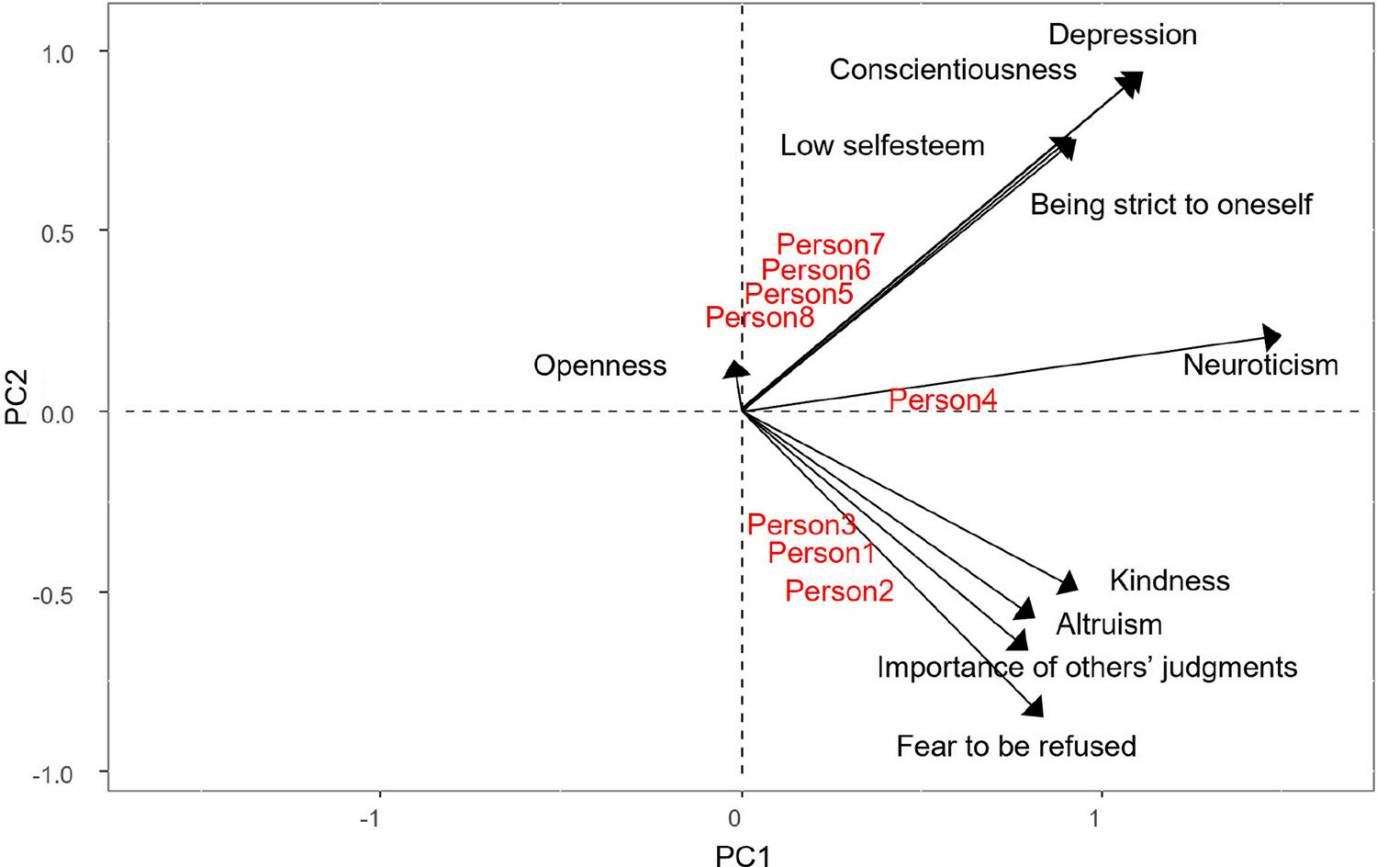
Biplot representation of persons and their dispositions

From Fig. 4, it can be seen that the personality traits of person1, person2, and person3 are mainly fear to be refused, kindness, importance of others’ judgments, and altruism. We could therefore describe them as other-oriented people. On the other hand, person5, person6, person7, and person8 are characterized by low self-esteem, conscientiousness, being strict to oneself, and depression, i.e., self-oriented people. Person4 is mainly neuroticist.

**Table 4:** Component values for the dispositions, resulting from the Tucker3-PCovR (values exceeding 0.4 in absolute value are bold

Disposition	Comp 1	Comp2
Fear to be refused	**0.84**	$\mathbf {-0.85}$
Kindness	**0.94**	$\mathbf {-0.50}$
Importance of others’ judgments	**0.80**	$\mathbf {-0.66}$
Altruism	**0.82**	$\mathbf {-0.58}$
Neuroticism	**1.50**	0.21
Openness	−0.02	0.14
Being strict to oneself	**0.92**	**0.76**
Low self-esteem	**0.93**	**0.76**
Conscientiousness	**1.09**	**0.92**
Depression	**1.12**	**0.93**

To interpret the relationships between the elements in different modes, which reflect the interactions between them, the core array **G** is needed (Table 5). Looking only the largest (in absolute value) core values, it appears that person1, person2, and person3 when faced with situations such as quarrelling with someone, partner leaves you, or someone is telling lies about you feel other anger, shame, sorrow and unloved. Remember that these people were characterized by their kindness, altruism, importance of others’ judgments and fear to be refused. On the contrary, person5, person6, person7, and person8 react to situations such as giving a bad speech, failing a test or writing a bad paper react with feelings of guilt and self-anger. It is important to note that these people are characterized by low self-esteem, conscientiousness, being strict to oneself, and depression.

**Fig. 5:**
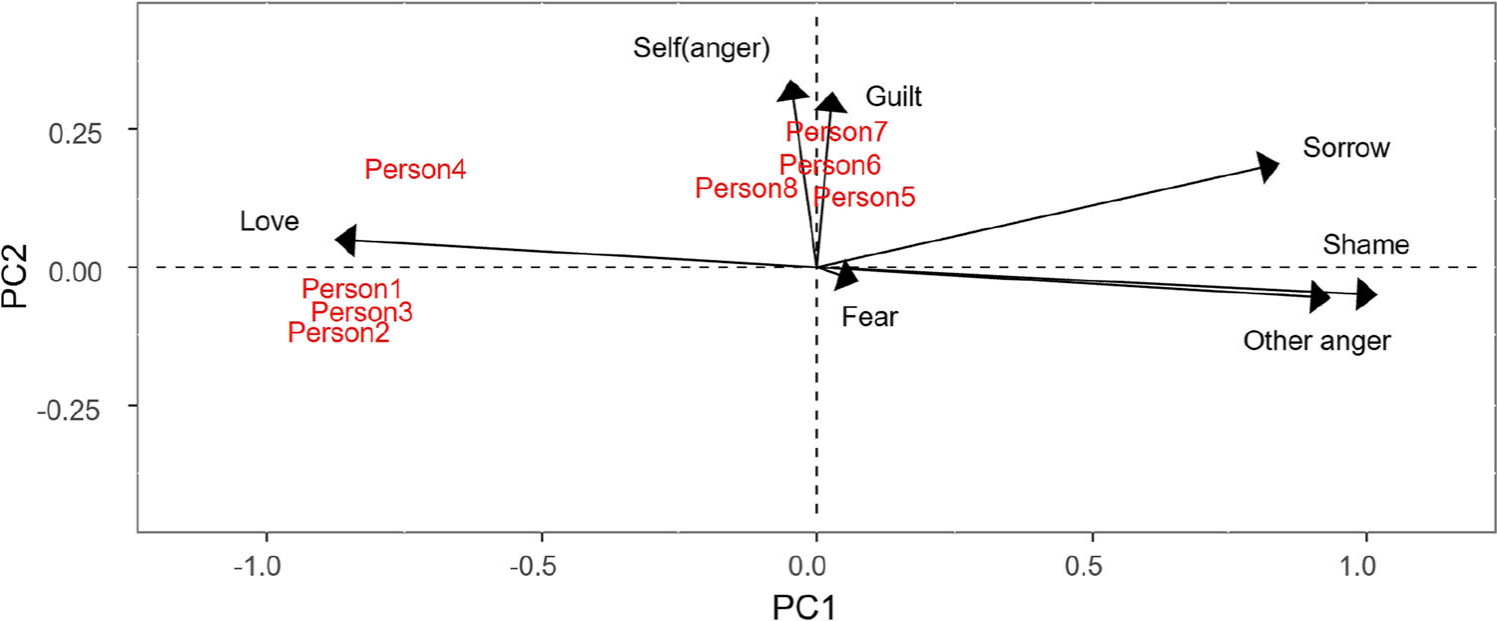
Joint biplot for persons and responses for the first component of situations

**Fig. 6:**
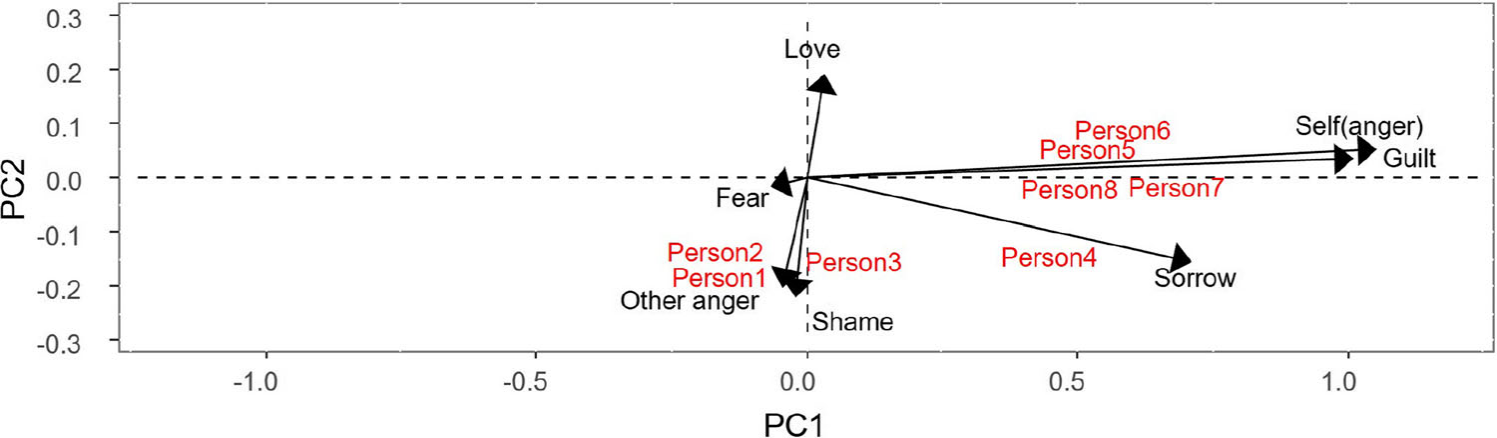
Joint biplot for persons and responses for the second component of situations

**Fig. 7:**
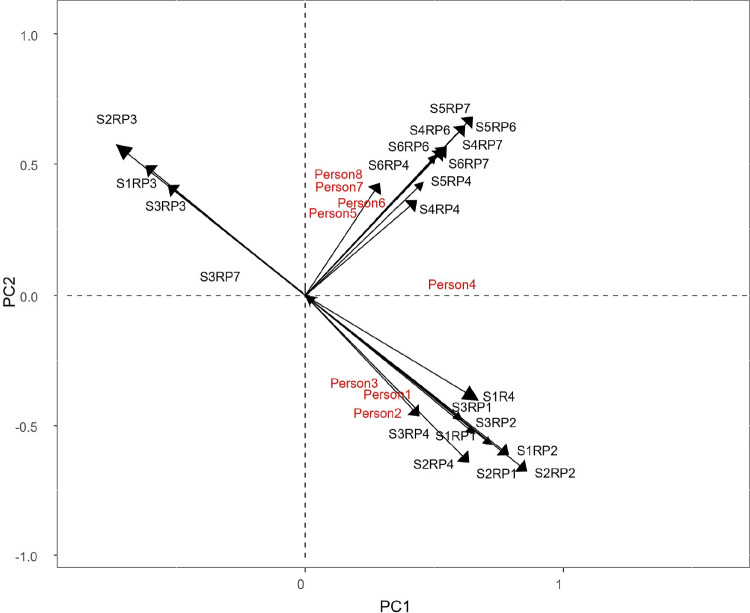
Interactive biplot

All this information can be extracted from the joint biplot (Figs. 5, 6) and the interactive biplot (Fig. 7).

Figure 5 shows the joint biplot for persons and responses for the first component of situations. This component takes negative values for quarrelling with someone, partner leaves you and someone is telling lies about you. This means that proximity between persons and responses in the graph is interpreted as a negative interaction and distance as a positive interaction for the above situations. On the other hand, love scores positively in this component, so the interpretation is the opposite. Figure 6, which shows the second component of situations, can be interpreted in the same way. Note that this component is positive for guilt and self-anger.

**Table 5:** Core array resulting from the Tucker3-PCovR analysis (values exceeding 1 in absolute value are in bold and values exceeding 2 in absolute value are underlined)

	Situation component 1		Situation component 2	
Person comp	Resp. comp1	Resp. comp2	Resp. comp1	Resp. comp2
1	**2.21**	0.62	−0.67	**1.45**
2	$\mathbf {-1.86}$	−0.22	−0.51	**1.57**

When analyzing the interactive biplot it must be taken into account that all combinations of situations and reactions are shown. From Fig. 7 it can be seen that all combinations of situations 1 (Quarrelling with someone), 2 (Partner leaves you) and 3 (Someone is telling lies about you) and reactions 1 (Other anger), 2 (Shame) and 4 (Sorrow) are negatively correlated with reaction 3 (Love) for the same situations and at the same time uncorrelated with combinations of situations 4 (Giving a bad speech), 5 (Failing a test) and 6 (Writing a bad paper) and reactions 4 (Sorrow), 6 (Guilt) and 7 (Self-anger). Obviously, the interpretation of the interactive biplot leads us to conclude the same results obtained previously.

**Fig. 8:**
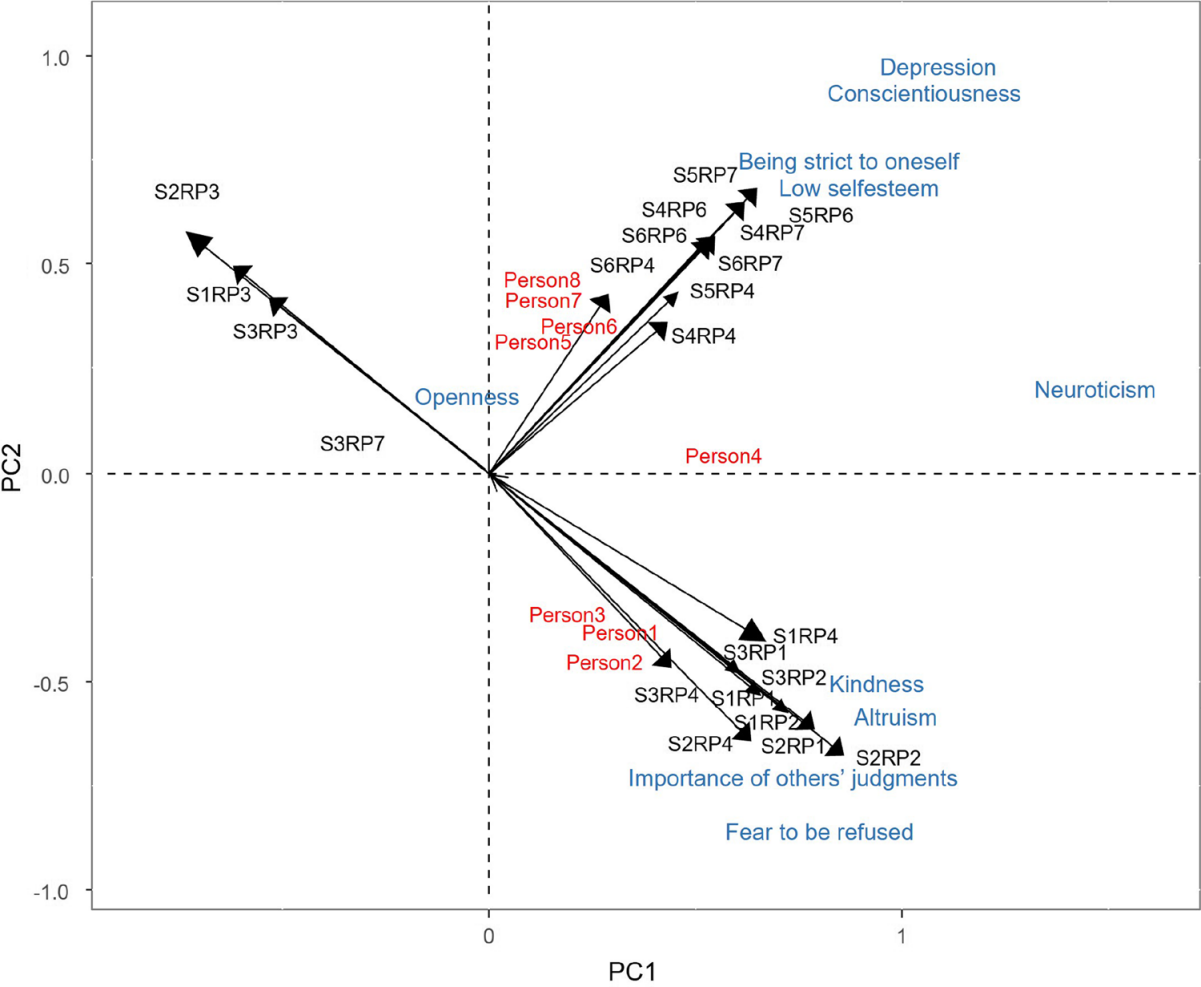
Simultaneous representation of persons, situations, reactions and dispositions

**Table 6:** Component values for the response scales, resulting from the Tucker3-PCovR analysis with $\alpha = 0.5$ (with values exceeding 0.20 in absolute value being indicated in bold)

Response	Label	Component 1	Component 2
Need to urinate frequently	RP1	$\mathbf {-0.21}$	0
Mouth gets dry	RP2	$\mathbf {-0.38}$	0
Need to defecate	RP3	$\mathbf {-0.35}$	**0.31**
Feel paralyzed	RP4	$\mathbf {-0.30}$	0
Full feeling in stomach	RP5	$\mathbf {-0.31}$	**0.29**
Perspire	RP6	$\mathbf {-0.35}$	−0.10
Feel nausea	RP7	$\mathbf {-0.20}$	$\mathbf {-0.22}$
Emotions disrupt action	RP8	−0.11	**0.41**
Heart beats faster	RP9	$\mathbf {-0.25}$	$\mathbf {-0.35}$
Feel exhilarated and thrilled	RP10	$\mathbf {-0.29}$	−0.14
Enjoy the challenge	RP11	$\mathbf {-0.24}$	0
Seek experiences like this	RP12	0	** 0.61**
Not want to avoid situation	RP13	0	0.14
Uneasy feeling	RP14	$\mathbf {-0.35}$	−0.18

It is also possible to create a “triplot” of people, situations, reactions and dispositions (Fig. 8) (captions in Figs. 7 and 8 represent all possible combinations of situations and responses. So *SiRPj* represents response $j-th$ and situation $i-th$). From Fig. 8 it is possible to predict how a person will react to a situation based on their personality traits. People with depressive, conscientiousness, being strict to oneself or low self-esteem personality traits, when confronted with situations such as giving a bad speech, failing an exam or writing a bad paper, experience reactions of sadness, guilt, and self-anger. On the other hand, people who have personality traits such as kindness, altruism, who value the judgements of others, or who are afraid of being rejected in situations such as quarrelling with someone, being abandoned by a partner, or having someone tell lies about you, experience other anger, shame or sadness. In addition, these people do not feel loved in these situations.

### The second example

A couple dataset was used, containing both the behavioral profiles and the dispositions of 128 participants. The S-R Inventory of Anxiousness (Endler & Hunt, 1968) scores of the 128 participants were collected in the three-way data array. This questionnaire measures 14 anxiety-related responses (Table 6) to 11 different stressful situations (Table 7) on a 5-point scale ranging from exhibiting the response “not at all” to “very much”. In addition, to measure personal dispositions the Cognitive-Affective Personality System questionnaire CAPS (Mischel & Shoda, 1995) was administered to each individual. This questionnaire consists of 63 items on a 7-point scale and it is designed to measure different types of cognitive-affective variables in relation to different types of stress. Therefore the Tucker3-PCovR analysis was performed on a three-way data array of size $\left( 128 \times 14 \times 11\right)$ and a data matrix of size $\left( 128 \times 63 \right)$ sharing the first mode (the 128 participants).

**Table 7:** Component values for the situations, resulting from the Tucker3-PCovR analysis with $\alpha = 0.5$ (with values exceeding 0.20 in absolute value being indicated in bold)

Situation	Comp 1	Comp 2
Speech before large group	0	$\mathbf {-0.20}$
Job interview	$\mathbf {-0.22}$	0
Match in front of audience	$\mathbf {-0.43}$	0
Consult counseling bureau	0	$\mathbf {-0.49}$
Ledge high on mountainside	0	−0.19
Sailboat on rough sea	$\mathbf {-0.53}$	0
New date	0.17	$\mathbf {-0.46}$
Auto trip	$\mathbf {-0.64}$	0
Alone in woods at night	0	$\mathbf {-0.41}$
Psychological experiment	−0.14	$\mathbf {-0.54}$

**Table 8:** Core array resulting from the Tucker3-PCovR analysis with $\alpha = 0.5$ (with values exceeding 20 in absolute value being indicated in bold)

	Situation component 1		Situation component 2	
Person component	Resp. comp1	Resp. comp2	Resp. comp1	Resp. comp2
1	$\mathbf {-27.41}$	1.94	$\mathbf {-61.46}$	**30.10**
2	**23.82**	−6.20	−0.48	−2.14

The three-way behavioral data array was pre-processed in such a way that the scores were mean-centered across persons and the scores for each response were normalized. The disposition data matrix was also pre-processed by standardizing.

Tucker3-PCovR analysis was performed on the previously pre-processed dataset for rank (2, 2, 2) and $\alpha = 0.50$ (same meaning for degree reduction and prediction). In this case, the choice of the rank was made in order to simplify the interpretation of the results obtained.

To obtain an easier to interpret solution, the response, situation and disposition component matrices were orthogonally rotated by varimax (Kaiser, 1958) and then the person component matrix and the core array were counterrotated.

The post-processed component matrices for the response scales and situations are given in the Tables 6 and 7. The post-processed core array is presented in the Table 8. In this table, the rows correspond to the person components and the columns to the combinations of a response and a situation component. Due to the size of the person component matrix, it is not included in the article.

Looking at the response component matrix (Table 6), one can see how some responses involving a physical manifestation load negatively on the first component (e.g., need to urinate frequently, mouth gets dry, feel paralyzed or perspire) while some others load negatively on the first component and positively on the second (e.g., need to defecate or full feeling in stomach). Feeling nausea and heart beats faster load negatively on both. Reactions related to seeking/avoiding the situation are distributed between the first and second components, e.g., emotions disrupt action load the second component while enjoying the challenge loads the first. Similarly, situations are distributed between the two components (Table 7), so that situations such as job interview or match in front of audience load the first component while new date or alone in woods at night load the second.

**Fig. 9:**
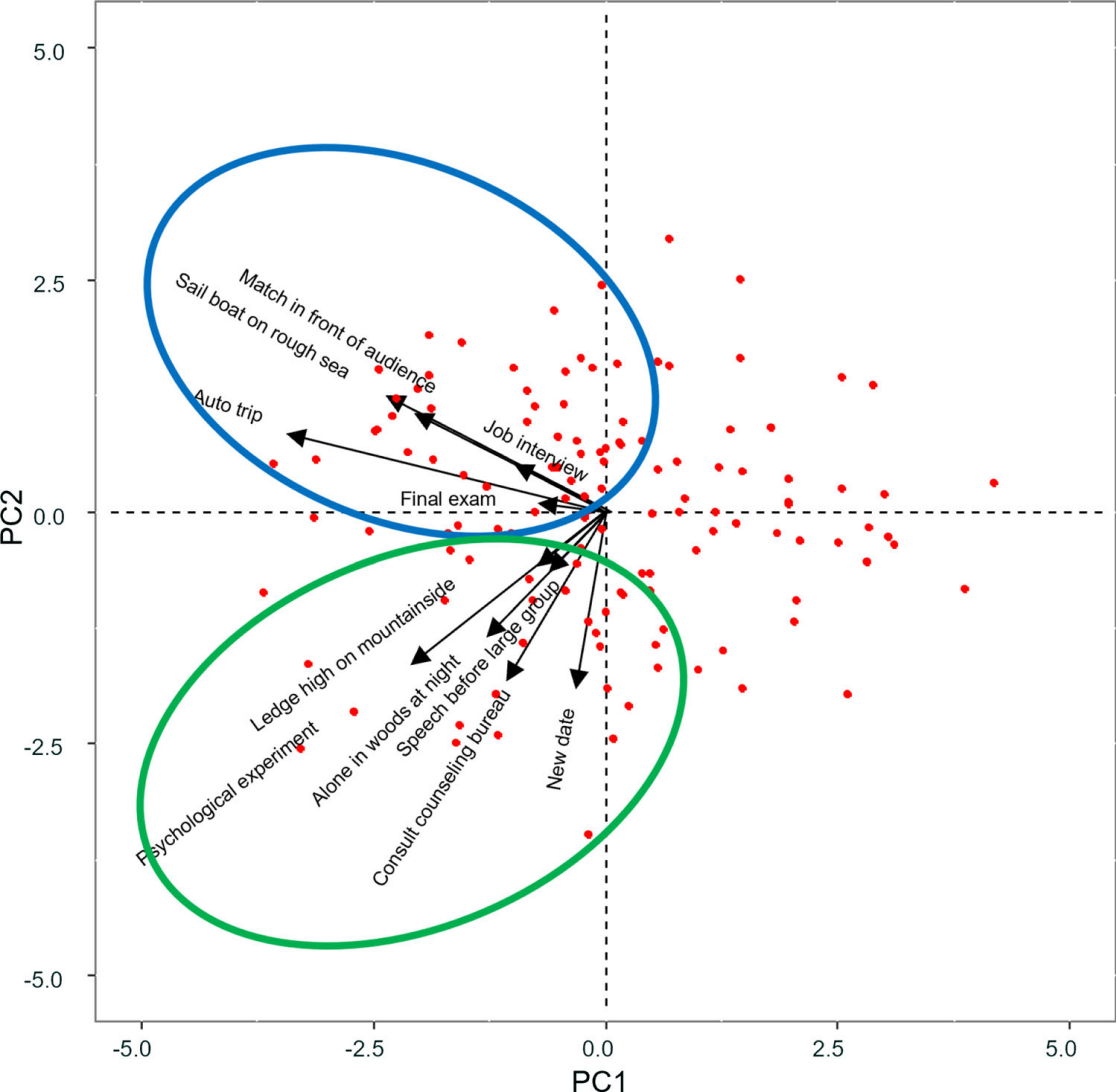
Joint biplot for persons and situations for the first component of responses

**Fig. 10:**
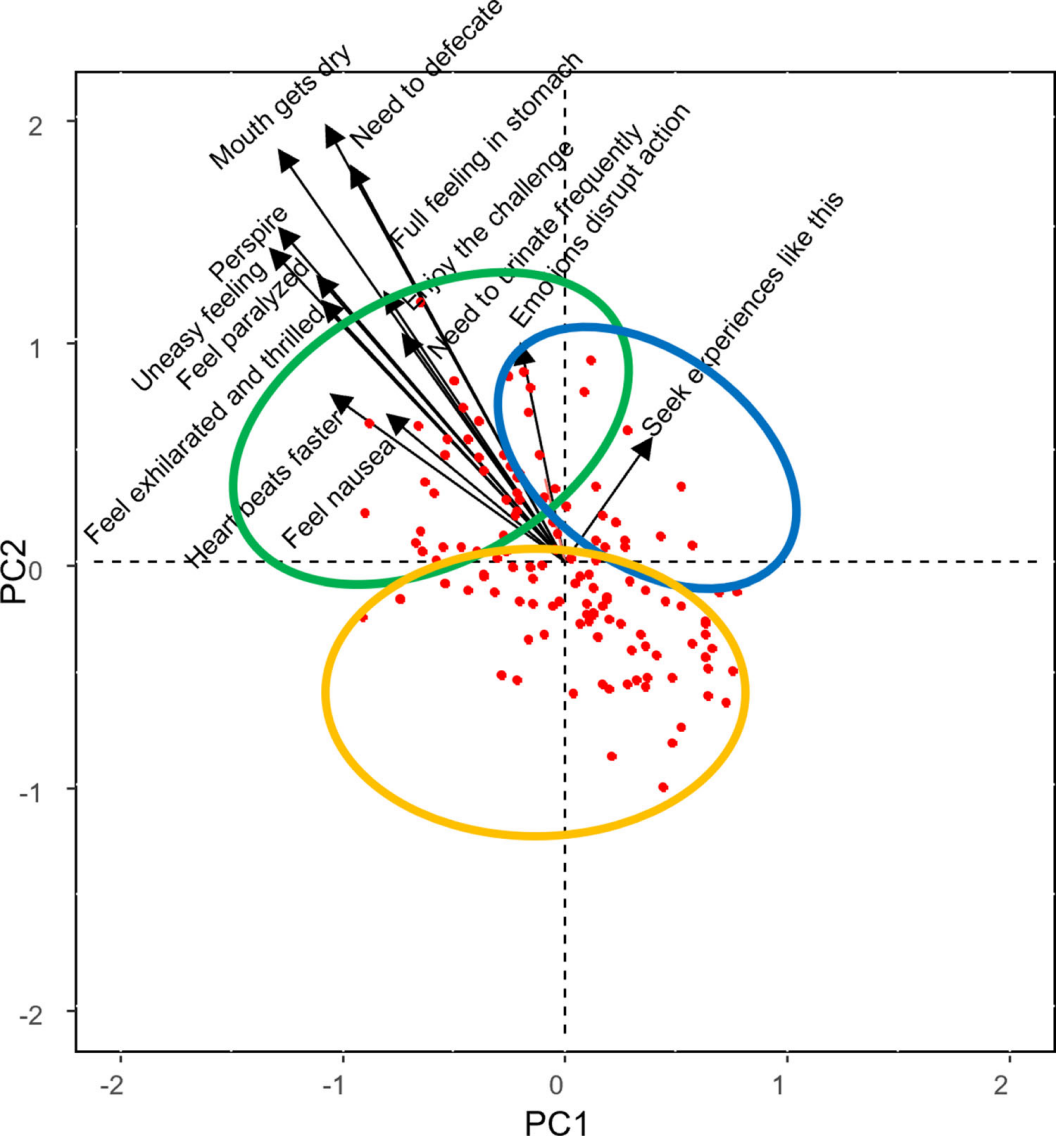
Interactive biplot for persons and responses for the situation “Match in front of audience”

The core array (Table 8) summarizes the main interactions between the different components corresponding to people, situations, and responses. When examining the core array only the largest (in absolute value) core values should be considered. From the core array, it can be deduced that people who score high on the first component will show first component reactions when faced with first component situations. For example, when faced with a job interview, these people will experience the following physical manifestations: need to urinate frequently, mouth gets dry, need to defecate, feel paralyzed, full feeling in stomach,... with higher or lower frequency than the average. On the other hand, in situations with the second component, they manifest all kinds of reactions above or below the average. In relation to people who have a high second component load in first component situations, they only show above or below average first component reactions.

More information can be obtained by analyzing biplots. Figure 9 shows the joint biplot for persons and situations for the first component of responses. We can see that there are two groups of situations. Situations such as match in front of audience, sailboat on rough sea, final exam, auto trip and job interview are highly correlated. The rest of the situations are highly correlated.

Given the large number of situations and responses, interactive biplots are created for two specific situations (each loading on a different component) to facilitate interpretation of the results. Figure 10 shows the responses to the situation “Match in front of audience” (which is highly loaded on the first component). It can be seen that some people experience different physical reactions and do not seek such experiences. Others score high on seeking such experiences and do not experience physical reactions. The rest of the individuals do not react in any way.

**Fig. 11:**
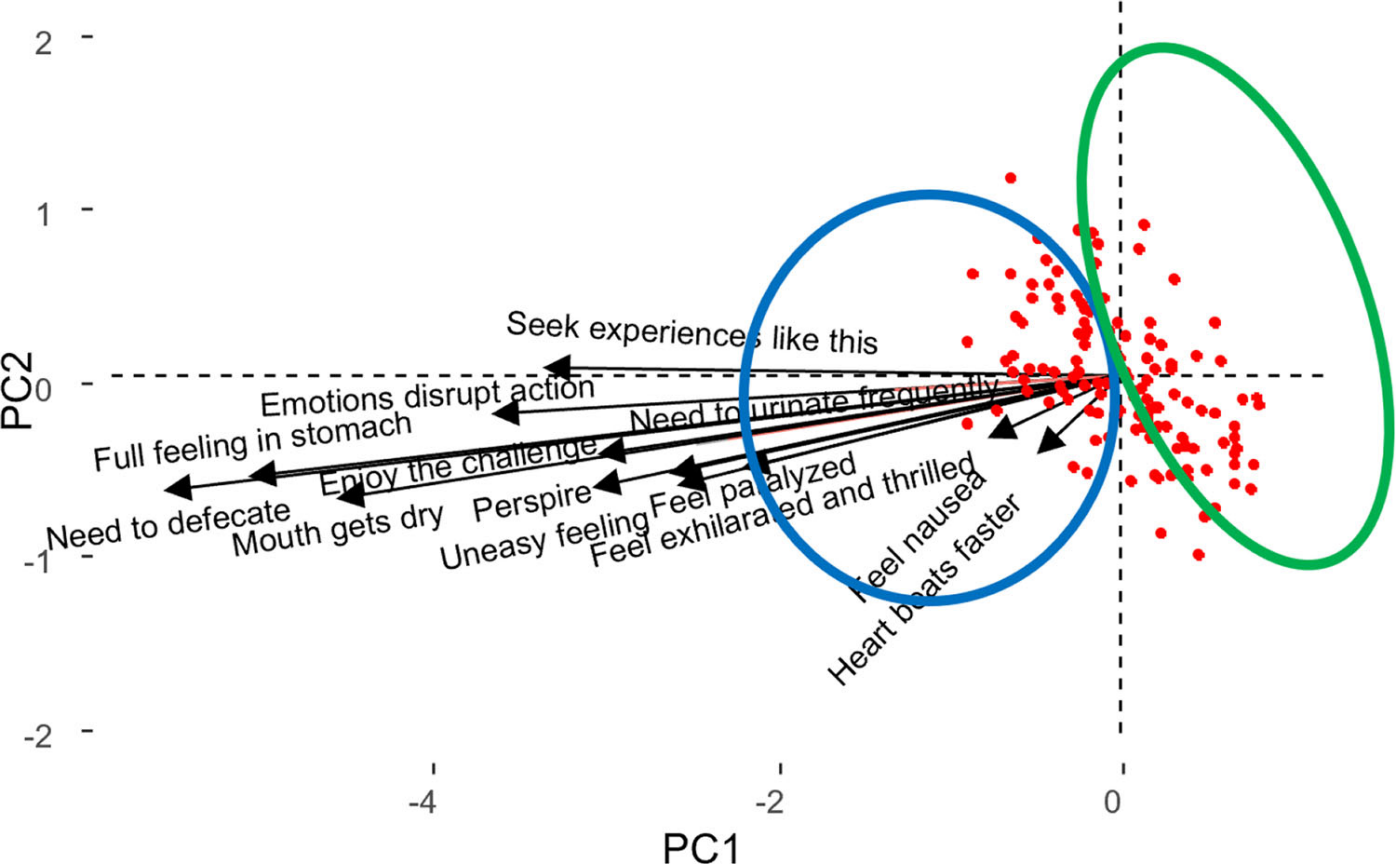
Interactive biplot for persons and responses for the situation “New date”

Looking at Fig. 11, which corresponds to the reactions to the “new date” situation (which is highly loaded on the second component), it can be observed that a group of people experience different physical reactions but do not want to avoid the situation, while others do not experience any reaction.

**Fig. 12:**
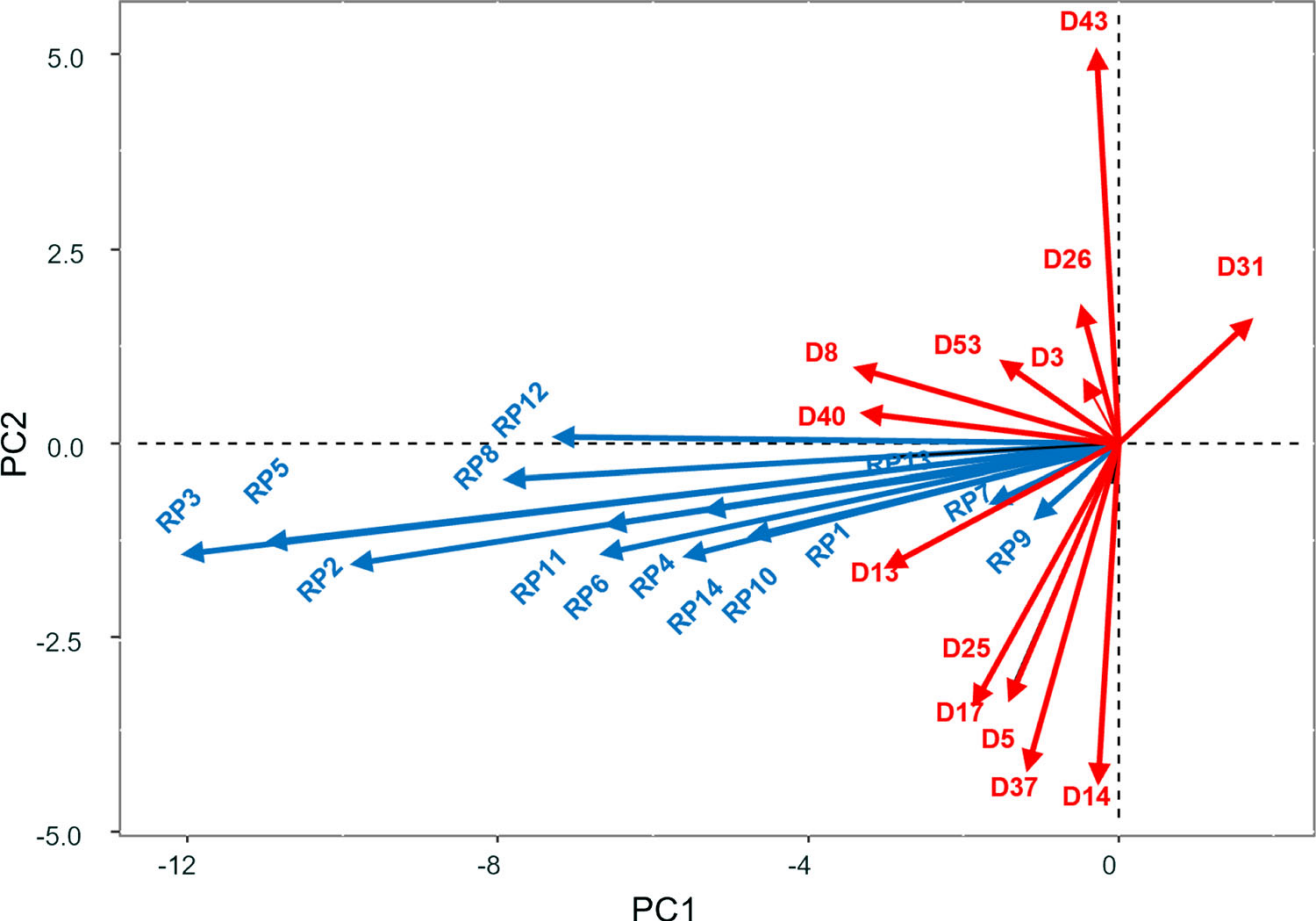
Simultaneous representation of reactions and dispositions for the situation “New date”

Furthermore, a triplot of responses and dispositions was made for the “new date” situation in order to analyze the relationship between them (Fig. 12). Given the large number of dispositions, it was decided to show only some of them. Descriptions of the disposition and response labels can be found in Tables 6 and 9, respectively.

In Fig. 12, we can see that the dispositions most correlated with the responses for the situation “New date” are “In a physically threatening situation I readily expect that my health will be damaged” (D8), “I easily experience a situation in which I may suffer physical injury, as risky” (D40) and “In an unclear situation, I immediately prepare myself for the worst” (D13). However, people with dispositions such as “I can deal well with stress” (D43) do not experience any of the above reactions (the angle formed by these vectors is straight). Relationships between dispositions could also be analyzed; for example, “I easily experience a situation in which I may suffer physical injury, as risky” (D40) and “I can deal well with stress” (D43) are uncorrelated and “When I am obliged to do something, I easily think that I am not able to do it” (D17) and “I often expect that things will end badly” (D5) are highly correlated. In summary, this figure shows the reactions to the ’new date’ situation in relation to the individual’s personality profile. The same analysis could be carried out for all other situations so that it would be possible to predict how a person will respond to a situation according to his or her personality profile.

## Conclusion

**Table 9:** Labels for (selected) dispositions used in the coupled dataset

Label	Description
D3	I think it is important to be healthy
D5	I often expect that things will end badly
D8	In a physically threatening situation I readily expect that my health will be damaged
D13	In an unclear situation, I immediately prepare myself for the worst
D14	I find it hard to stay calm in a stressful and uncertain situation
D17	When I am obliged to do something, I easily think that I am not able to do it
D25	When I fail, I am completely thrown off balance
D26	I think it is important to keep my body fit
D31	I can cope with a physically threatening situation
D37	In a performance-related task, I easily expect that I will fail
D40	I easily experience a situation in which I may suffer physical injury, as risky
D43	I can deal well with stress
D50	I think it’s important to live without stress
D52	I attach great importance to having as little stress as possible
D53	I tend to pay much attention to physiological changes in my body

This paper presents the Tucker3-PCovR model. It is a global model for the simultaneous analysis of a coupled dataset consisting of a three-way data array sharing a mode with a two-way data matrix in such a way that the data matrix is used to predict the data array. The goal of this model is to reduce the predictors to a few components that are used to predict the criterion. To achieve this, a global objective function is used that depends on a weight parameter, this weight indicates the importance that degree reduction and prediction have in the analysis.

We focus on the particular case where the three-way array is modeled by the Tucker3 model. An algorithm is proposed to implement the Tucker3-PCovR model. Moreover, given the difficulty in interpreting the results obtained, different biplots are proposed. More specifically, the joint and the interactive biplot are proposed for the visualization of the three-way data array while the triplot is suggested for the prediction of the three-way data array from the two-way data matrix. Finally, the proposed model has been applied to analyze some datasets in psychology.

Both the method and the biplot representation are implemented in **R** (R Core Team, 2023). They are available as part of the R package *MultBiplotR* (Vicente-Villardon et al., 2023).

## Figures and Tables

**Algorithm 1:**
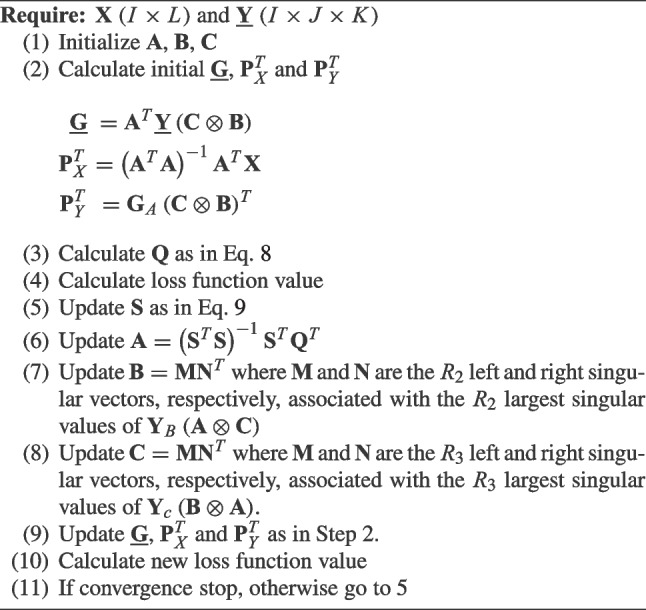
Tucker3 PCovR.

## Data Availability

The first dataset analyzed during the current study is available in the GitHub repository in Tucker3-PCovR (https://github.com/efb2711/Tucker3PCovR).

## A. Appendix

The ALS algorithm to perform the proposed Tucker3 PCovR is the following:

## Code Availability

The software to perform Tucker3-PCovR analysis is available in the **R** (R Core Team, 2023) package *MultBiplotR* (Vicente-Villardon et al., 2023).
